# Early failures when using three different adhesively retained core build-up materials—a randomized controlled trial

**DOI:** 10.1007/s00784-021-04170-6

**Published:** 2021-09-07

**Authors:** Andreas Zenthöfer, Justo Lorenzo Bermejo, Wolfgang Bömicke, Cornelia Frese, Rumeysa Gülmez, Peter Rammelsberg, Brigitte Ohlmann

**Affiliations:** 1 Department of Prosthodontics, Dental School, University of Heidelberg, Im Neuenheimer Feld 400, 69120 Heidelberg, Germany; 2 Institute of Medical Biometry, University of Heidelberg, INF 130.3, 69120 Heidelberg, Germany; 3 Department of Operative Dentistry, Dental School, University of Heidelberg, Im Neuenheimer Feld 400, 69120 Heidelberg, Germany

**Keywords:** RCT, Clinical, Core build-up, Failure, Dental prostheses, Adhesive

## Abstract

**Objectives:**

To compare the failure rates for three different adhesively retained core build-up composites up to the incorporation of a permanent fixed dental prosthesis (FDP), and to identify potential failure risk factors.

**Material and methods:**

A randomized controlled trial of 300 participants in need of a core build-up to restore a vital abutment tooth before prosthetic treatment was conducted. Participants were assigned by stratified block randomization to one of three study groups: Rebilda DC (RDC), Clearfil DC Core (CDC), or Multicore Flow (MF). Test teeth were prepared by use of the respective manufacturer’s adhesive system. The total-etch technique was used for RDC and MF, and the self-etch technique for CDC. Participants were treated by dentists (*n* = 150) or dental students (*n* = 150). Failure rates of core build-ups before incorporation of FDPs were investigated using univariate and multiple logistic regression.

**Results:**

The overall failure rate was 8% (*n* = 23). Rate differences between the three investigated groups did not reach statistical significance (*p* > 0.05). The mean time between placement of core build-ups and placement of fixed dental prostheses was 12.2 (SD: 14.2) weeks. Conversely, larger cavities (> 3 surfaces) and treatment by dental students were independently associated with an increased failure risk (*p* < 0.05).

**Conclusions:**

The main risk factors for early failure seem to be the size of the core build-up and clinical experience of the operator, whereas failure rates of core build-up materials combined with a self-etch approach seem to be similar to the rates of materials combined with the total-etch technique.

**Clinical significance:**

This research article should give clinicians an impression of the short-term performance of different adhesively retained core build-ups using different adhesive techniques/materials. Moreover, predominant influencing factors for the success or failure should be pictured.

## Introduction

The replacement of decayed dental hard tissues by means of a core build-up is an important work step during the prosthetic management of single crowns and fixed dental prostheses [1–3]. The retentive preparation design required for prosthetics can often only be achieved by fabricating a core build-up. Ideally, the material used should ensure its stable retention in the tooth stump. It should also be grindable, similar to dentin, and distinguishable from the tooth structures by its opacity. Adhesively retained resin composite materials sufficiently fulfill these requirements and are state-of-the-art [4–11]. However, if resin composite materials are used for teeth with larger subgingival defects being prepared for fixed dental prostheses, moisture control and tissue management can be difficult [12]. Several studies have indicated that use of the total-etch technique achieves the most reliable bonds to dental hard tissue in permanent direct composite resin restorations, including the application of a hydrophilic primer and hydrophobic layer of bonding [13–16]. However, creating adhesion—especially when done using multiple work steps—is very technique-sensitive [17]. With regard to core build-ups fitted in a clinical setting, one study revealed that a not insignificant proportion of composite core build-ups fails before incorporation of the final dental prosthesis, although a significantly higher incidence of failure was recorded for glass-ionomer cements than for composites [18]. In contrast, another clinical study found no early failures for adhesively retained core build-ups [19]. Nonetheless, various determinants can impact the success of core build-ups, i.e., tooth location, presence of ferrule design, and cavity size [1, 2, 20]. Back to adhesion: in modern dentistry, simplification of the technique-sensitive bonding procedure by means of reduction of components/work steps up to all-in-one adhesives (self-etching and priming adhesives) is becoming increasingly popular [13–17]. In this technique, acidic monomers modify the smear layer and simultaneously etch and prime the dental hard tissues. It has been suggested that reduced penetration of the dentin’s tubular structure decreases postoperative sensitivity; however, it also results in reduced bond strength [14, 15]. Another substantial drawback of some self-etch adhesives is that they can inhibit complete polymerization of self- and dual-curing composite materials [21, 22]. This problem has been recognized by the industry, which has focused on improving the compatibility of corresponding adhesive systems [23]. Nonetheless, no information is available regarding the clinical performance of resin core build-ups used in combination with self-etch adhesives. In contrast to permanent restorations, core build-ups are only in direct contact with the oral cavity until the final dental prosthesis is fitted [11]. Provided the performance of resin core build-ups is comparable, the use of these materials might reduce treatment time and would probably be less technique-sensitive. Therefore, the objective of this randomized controlled trial was to evaluate the incidence of early failure for three different adhesively retained resin core build-ups—including one material used with a self-etch system—and to identify possible risk factors for failure before incorporation of the permanent prosthetic restoration. In addition, the possible influence of the operator (dentist vs student) should be evaluated. The null hypotheses were (1) the short-term performance of the three materials would not differ and (2) the short-term performance of core build-ups done by dentists and students is likewise.

## Materials and methods

### Setting/participants

This randomized controlled trial was approved by the local ethics committee of the University of Heidelberg (registration no. S-112/2011), and the study protocol was also registered at clinicaltrials.gov (registration no. NCT01449903). The study was conducted mono-centrically among patients visiting the Department of Prosthodontics at Heidelberg University. Assuming failure rates of 10 (Multicore Flow), 20 (Rebilda DC), and 30% (Clearfil DC Core) in the three treatment groups, a sample size calculation revealed that 100 participants per group (300 in total) are needed to detect a different failure rate in at least one of the investigated groups with 80% statistical power (*α* = 5%; uncorrected chi-squared test). The assumed failure rates were based on a study from 2005 [18] and own pre-investigation data. To meet the study inclusion criteria, participants had to be fitted with a single crown or fixed dental prosthesis (FDPs) and be in need of abutment tooth reconstruction with a core build-up. Treatment could take place either during the clinical students’ course or the dentists’ consultation hour at the department. Only one restoration per participant was considered. The study tooth had to be vital and free from complaints. If more than one tooth was suitable for inclusion, the study tooth was selected according to the Féderation Dentaire Internationale (World Dental Federation, FDI) tooth scheme (teeth 18–48); e.g., if both tooth 16 and tooth 14 needed core build-ups, tooth 16 was assigned as study tooth because 16 orders first according to FDI numbering (16, 15, 14, 13 …). If the test tooth required more than one core build-up, only the largest cavity (largest coherent volume) was included in the study (e.g., not the small cervical lesion was considered). Participants were also required to sign an informed consent form, be at least 18 years of age, and have no intention of moving house or changing dentist during the study period. Pregnant and nursing women and patients with poor oral hygiene or known allergies to the study materials were also excluded from participating.

### Randomization

**Fig. 1:**
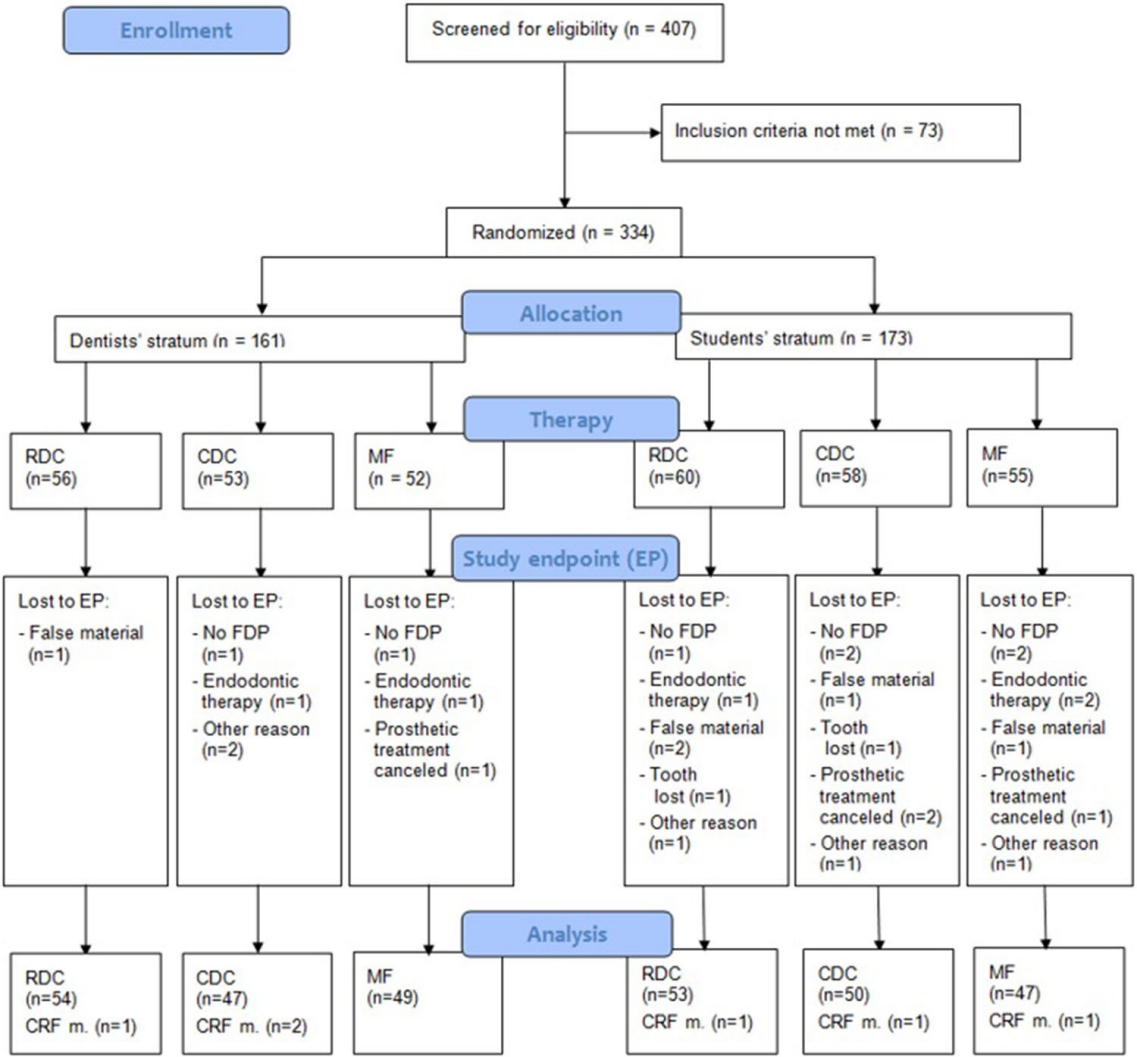
Overview of study progression. RDC, Rebilda DC; CDC, Clearfil DC Core; MF, Multicore Flow; FDP, fixed dental prosthesis; CRF, case record form; CRF m., missing CRF. For better visualization of the strata, “tooth location” is not depicted. It includes three sub-arms (incisor, premolar, and molar) and is located between the operators’ stratum and the three different core build-up materials

A combined eligibility-participation rate of about 75% was assumed and in total, 407 potential patients were examined for eligibility during regular dental treatment at the department. Seventy-three patients did not meet the inclusion criteria or refused to participate. Of the finally included patients, 34 participants were recruited in the further course of the study to compensate for drop-outs and to meet the required number of cases according to the sample size analysis, resulting in 300 study participants available for statistical analysis at the endpoint. Participants were assigned to treatment groups by means of stratified block randomization, applied separately to dental students and dentists (first stratum) and tooth location (anterior, premolar, or molar; second stratum). Each participant was assigned to one of the three study groups by drawing lots: (1) Rebilda DC white/Solobond Plus (RDC, Voco GmbH; Cuxhaven, Germany), (2) Clearfil DC Core (Plus) white/Clearfil DC Bond (CDC, Kuraray Europe GmbH; Hattersheim, Germany), (3) Multicore Flow/Syntac (MF, Ivoclar Vivadent AG; Schaan, Liechtenstein). The principal investigators (AZ/BO) were informed by the operator when a patient was willing to participate in the study and met inclusion criteria. AZ/BO draw the lot and the operator was informed about the material assigned. The patient was blinded against the lot decision while the operator could not. Figure 1 provides an overview of the study’s progression.

### Clinical fabrication procedures

**Fig. 2:**
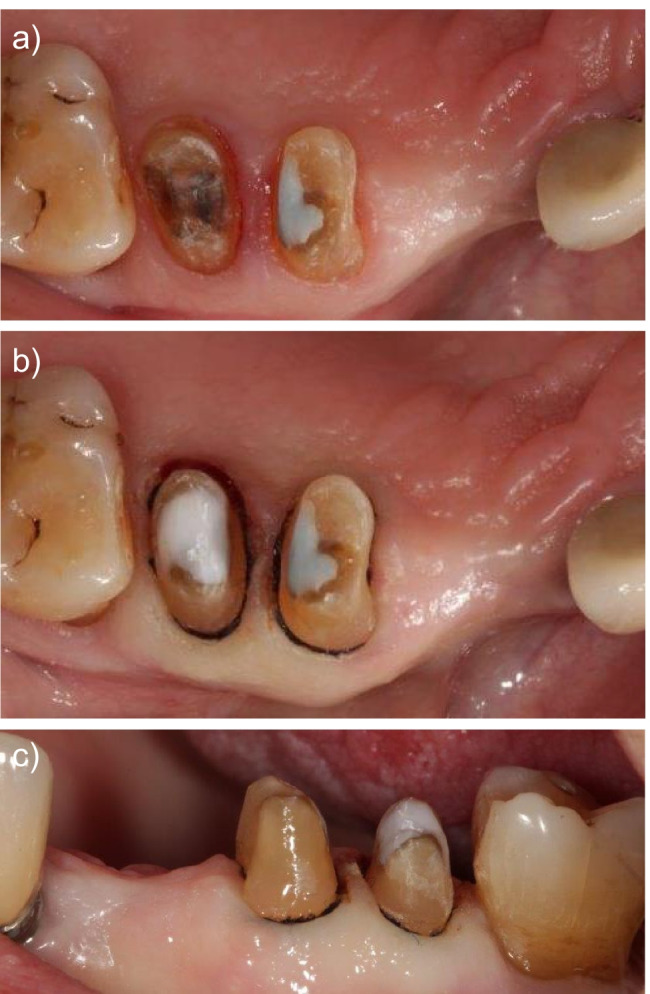
Exemplary clinical situation. **a** Tooth 15 after removal of the insufficient restorations and caries excavation. **b** Tooth 15 fitted with a core build-up, occlusal view. **c** Same situation lateral view

**Fig. 3:**
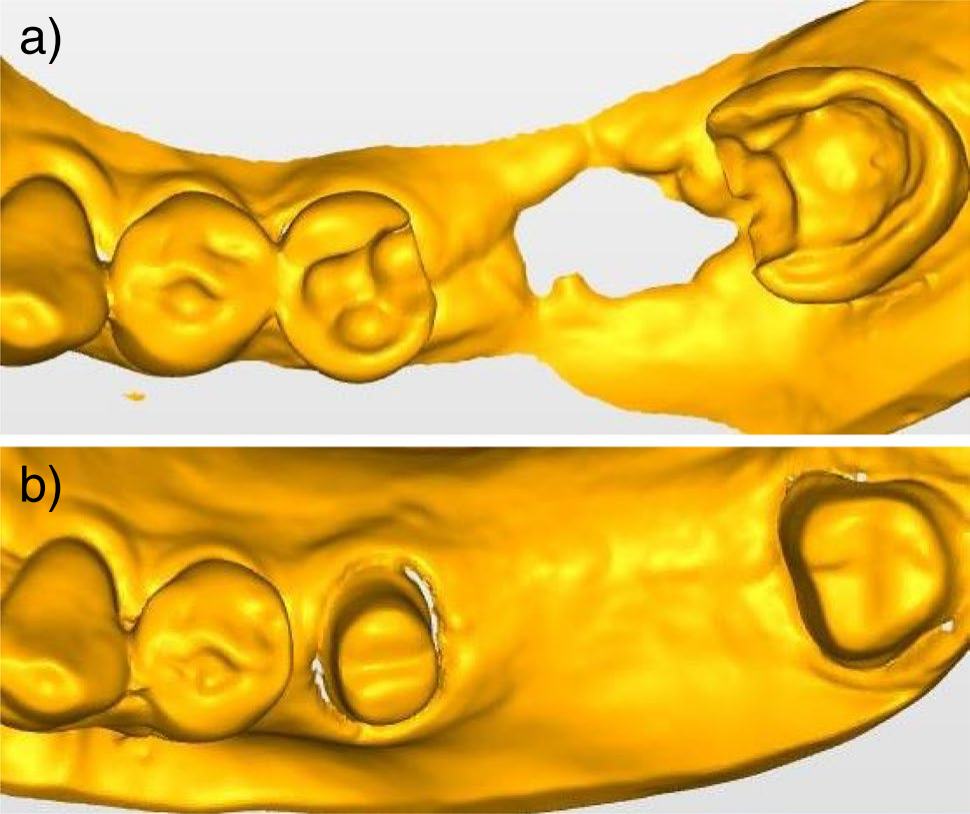
Exemplary clinical situation from the study. **a** Scan of the excavated teeth 37 and 35. **b** Scan after fitting with core build-ups (tooth 37 was considered for the study according to FDI scheme)

See Fig. 2a–c for an exemplary clinical case. Another case is visualized in Fig. 3.

Dental treatment was performed according to the standard guidelines for operative and prosthetic dentistry. If needed, inadequate prostheses were removed and caries completely excavated. Afterwards, the cavity was cleaned by use of 0.1% chlorhexidine rinse. A rubber dam or swabs, matrices, and retraction cords were used for tissue and moisture control if required. At least relative moisture control by the use of a saliva ejector, swabs, and retraction cord was mandatory. Cavities were prepared according to randomization (see Fig. 1) by means of the adhesive systems corresponding to the resin core build-up materials. The exact procedure for each material is described below. Core build-ups were inserted using automix syringes (each only one increment). A chamfer design incorporating a ferrule of at least 1.5 mm was used to prepare the study teeth for full crowns (for anchorage of single crowns or FDPs). Teeth were fitted with temporary composite resin-based restorations (Luxatemp Solar; DMG, Hamburg, Germany) and impressions taken using a polyether material (Impregum; 3 M, Seefeld, Germany). Participants were scheduled for their placement appointment (study endpoint) and advised to contact their study dentist if the temporary restoration failed or another noteworthy event occurred in relation to the test tooth in the meantime. Provisionals were removed by use of arterial clamps or scalers. At the placement appointment, the respective restorations—which were produced by local dental laboratories—were tried in the participant. These were adjusted and polished if necessary before finally being incorporated (endpoint of the study). Completely failed core build-ups were replaced by use of the RDC approach (adhesive: OptiBond FL). Partially failed core build-ups were repaired subsequentially using the local standard procedure including intraoral sandblasting and silanization.

### Core build-up materials and their application

#### RDC

The cavity was etched using 35% orthophosphoric acid starting at the enamel margins (etching time of 30 and 15 s for enamel and dentin, respectively), and then rinsed with water spray to remove the etching agent. The cavity was air-dried and the primer gently brushed over the dentin surfaces for 30 s using a microapplicator before subsequently being dried. A thin layer of adhesive was applied to the dentin and enamel for at least 15 s and light cured for 20 s. Finally, RDC was applied and cured for 60 s.

#### CDC

Liquid A und liquid B of the self-etch adhesive system were mixed in the ratio 1:1 for 5 s and brushed in the cavity (enamel and dentin) for 20 s. The manufacturer’s instruction did not require selective application of phosphoric acid for the enamel. To remove the solvent agent, the cavity was dried under a gentle stream of air. The adhesive was cured for 20 s. Finally, CDC was applied to the cavity and light cured for 60 s.

#### MF

The cavity was etched using 37% orthophosphoric acid (30 and 15 s for enamel and dentin, respectively). The etching agent was then removed using water spray (45 s). The primer was brushed over the dentin surface for 15 s using a microapplicator and dried under a gentle stream of air. Adhesive I (Syntac) was applied to the cavity for 15 s and dispersed under a gentle stream of air. Adhesive II (Heliobond) was then applied, and the adhesives were cured for 20 s. The core build-up material MF was inserted into the cavity and cured for 40 s.

The core build-ups were finished and polished or immediately prepared for a full crown. Light curing of all materials was performed using a polymerization lamp with a radiance of 1200 W/cm^2^ (Elipar S10; 3 M Oral Health Care, Seefeld, Germany).

### Target variables

The main target criterion was survival of the core build-up of the test tooth until incorporation of the permanent prosthetic restoration (depending on the indication (e.g., implants involved in restoration) and extent of treatment needed this time varied, see “Results”). If a core build-up failed, the reason for this was noted. The following details were recorded on case record forms for each participant at baseline (placement of core build-up): study tooth, tooth region, core build-up material used, number of restored surfaces, and moisture control. In addition, the dentist or dental student in charge rated the handling properties and grindability of the materials on a scale of 0–10, where 0 = poor and 10 = very good. At the incorporation appointment (study endpoint), the number of impressions taken and number of changes to the temporary restoration were also recorded.

### Statistical analysis

Means, standard deviations (SDs), and frequencies (%) were calculated for descriptive purposes. The characteristics of the participants treated by dental students and dentists were examined separately (first stratum). Differences between incidences of failure were analyzed using chi-squared tests, and average operator ratings (grindability and handling properties of the core build-up materials) were compared using *t*-tests. For statistical testing, continuous variables were grouped according to sample medians or clinical relevance. The incidence of failure for core build-ups during the study period was calculated separately for the following variables: gender, age, core build-up material, tooth region, operator (dentist/student), number of impressions taken, and number of changes to the temporary restoration. Differences in failure rates were investigated by univariate logistic regression considering the failure of the core build-up (yes = 1; no = 0) as the dependent variable and the investigated factors as explanatory variable. The factors associated with failure rates in the univariate analyses (*p* < 0.05) were simultaneously considered in a multiple logistic regression model. In addition to the overall analysis, separated models were fitted the dentist and the dental student data. All odds ratios were interpreted as relative risk estimates. Statistical analysis was performed by use of SPSS v19.0 (IBM; New York, USA); *p* values < 0.05 were declared to be statistically significant.

## Results

### Study population

Table 1 provides general data and a comparison of variables according to the main stratum (students and dentists). In total, 300 of the 334 participants initially included in the study reached the endpoint (drop-out incidence: 10.2%). The reasons for the 34 drop-outs are given in Fig. 1. The average age of the study population (*n* = 300) was 59 years (SD: 11 years) and nearly half of participants were female (49%). Half of the participants were treated by dentists (*n* = 150). The mean time between placement of core build-ups and placement of fixed dental prostheses was 12.2 weeks (SD: 14.2; min: 0; max: 97). In the dentist and student group, respectively, mean time interval was 14.3 (SD: 17.4; min: 0; max: 97) and 10.1 (SD: 9.8; min: 0; max: 54) weeks. Most core build-ups were placed in the posterior region (88%). With respect to the core build-up material used, 107 participants (36%) received RDC, 97 (32%) CDC, and 96 (32%) MF. The dental students changed the temporary FDPs more frequently than the dentists did and needed more attempts to take an adequate impression (*p* = 0.001). Rubber dam was more frequently used by the dental students (p < 0.001). In terms of operator ratings, dental students rated the handling properties of the core build-up materials one unit worse, on average, than dentists did (*p* = 0.001). Apart from these findings, the characteristics of patients treated by dental students and dentists were comparable (*p* > 0.05).

**Table 1:** Participant characteristics and target variables, separated for the main stratum (treatment by a dentist or student) (*n* = 300)

Variable	Dentist (*n* = 150)	Student (*n* = 150)	Complete cohort	*p* value
Patient age, mean (SD)	59.9 (11.9)	57.8 (10.6)	58.9 (11.3)	0.11
Patient gender, number (%)				
Female	71 (47.3)	75 (50.0)	146 (48.7)	0.64
Male	79 (52.7)	75 (50.0)	154 (51.3)	
Tooth region, number (%)				
Incisor	22 (14.7)	15 (10.0)	37 (12.3)	
Premolar	58 (38.7)	66 (44.0)	124 (41.3)	0.40
Molar	70 (46.7)	69 (46.0)	139 (46.3)	
Material, number (%)				
Rebilda	53 (35.3)	54 (36.0)	107 (35.7)	
Clearfil	50 (33.3)	47 (31.3)	97 (32.3)	0.93
Multicore	47 (31.3)	49 (32.7)	96 (32.0)	
Surfaces, mean (SD)	2.8 (1.1)	2.9 (1.2)	2.8 (1.3)	0.21
Impressions, mean (SD)	1.5 (0.8)	2.4 (1.6)	2.0 (1.3)	**0.001**
Change of temporary FDP, mean (SD), *n* = 292	2.2 (1.2)	3.4 (2.1)	2.8 (1.1)	**0.001**
Handling of material, mean (SD), *n* = 292	8.2 (1.9)	7.4 (2.1)	7.8 (2.0)	**0.001**
Moisture control (%)				
Rubber dam	6 (4.1)	32 (21.9)	38 (12.9)	
Relative	141 (95.9)	115 (78.2)	256 (87.1)	**0.001**
Grindability of material, mean (SD), *n* = 292	8.6 (1.7)	8.4 (1.5)	8.5 (1.6)	0.486

*p* values from chi-squared (discrete variables) or *t*-tests (continuous variables). *p* values < 0.05 are in bold

### Descriptive and univariate analysis of failures

Results from the univariate analysis of failures are shown in Table 2. During the study, 23 (8%) core build-ups failed. The vast majority of these failures occurred during removal of the temporary crown (78%). One failure occurred during impression taking (4.3%), three during preparation (13%), and a further failure occurred due to non-hardened material. Accounting for 18 (12%) failures during the study period, the participants treated by dental students experienced more failures than those treated by dentists (incidence of failure 3.3%; *p* = 0.008). Of the 23 failures, 15 occurred among core build-ups of > three surfaces, significantly more than among core build-ups of ≤ three surfaces (*p* = 0.0001). No failures were observed among the 28 patients treated using rubber dam moisture control. Differences between the incidence of failure for the other variables investigated did not reach statistical significance (*p* > 0.05).

**Table 2:** Univariate analysis of risk factors for early failures (*n* = 300)

Variable	Sub-variable	*n*	Failures	%	*p* value	OR	95%	CI
Gender	Female	146	12	8.2	0.73	Ref		
	Male	154	11	7.1		0.86	0.37	2.01
Age	23–59	154	11	7.1	0.73	0.86	0.37	2.01
	60–86	146	12	8.2		Ref		
Material	Rebilda	107	7	6.5	0.48	Ref		
	Clearfil	97	6	6.2		0.94	0.31	2.91
	Multicore	96	10	10.4		1.66	0.61	4.55
Tooth region	Incisor	37	1	2.7	0.14	Ref		
	Premolar	124	14	11.3		4.58	0.58	36.1
	Molar	139	8	5.8		2.20	0.27	18.2
No. surfaces	1–3	217	8	3.7	**0.0001**	Ref		
	> 3	83	15	18.1		5.76	2.34	14.2
Operator	Dentist	150	5	3.3	**0.008**	Ref		
	Student	150	18	12.0		3.96	1.43	11.0
Change of temporary restoration	0–2	158	16	10.1	0.10	2.17	0.87	5.44
	> 2	142	7	4.9		Ref		
Moisture control	Rubber dam	38	0	0.0	0.054	–	–	–
	Relative	256	23	7.8		Ref		

*p* values are based on univariate logistic regression analysis. Significant *p* values are in bold. *Ref.*, reference category; *OR*, odds ratio (relative risk estimate); *95% CI*, 95% confidence interval

### Multiple regression analysis of failures

Results from the multiple regression analysis of failures are shown in Table 3. Simultaneous consideration of the risk factors “number of surfaces” and “type of operator” confirmed the univariate results. The relative risk of failure was 3.7 times higher in participants treated by dental students compared to those treated by dentists. An increased failure risk was also found for core build-ups with more than three surfaces compared with the reference category (OR = 5.54; *p* = 0.0002). The proportion of core build-ups with more than three surfaces was 24.7% in the dentist group and 30.1% in the dental student group.

**Table 3:** Multivariate regression analysis of risk factors for early failure (*n* = 300)

Variable	Sub-variable	*n*	Failures	%	*p* value	OR	95%	CI
Surfaces	1–3	217	8	3.7	**0.0002**	Ref		
	> 3	83	15	18.1		5.54	2.22	13.8
Operator	Dentist	150	5	3.3	**0.01**	Ref		
	Student	150	18	12.0		3.75	1.33	10.6

*p* values are based on multivariate logistic regression analysis with significant independent variables from univariate analysis. Significant *p* values are in bold. *Ref.*, reference category; *OR*, odds ratio (relative risk estimate); *95% CI*, 95% confidence interval

### Operator ratings

Table 4 shows detailed ratings for the handling properties and grindability of materials, separated for dentists and dental students. The average score for the handling of core build-up materials (scale 0–10) was 7.6 for RDC, 7.9 for CDC, and 7.4 for MF. For grindability, the average ratings were 8.7 for RDC, 8.6 for CDC 8.6, and 8.2 for MF.

**Table 4:** Operator ratings for grindability and handling properties of the core build-up materials, separated for dentists and students (*n* = 292)

Rating	Dentists	*p* value	Students	*p* value
Handling of material				
1. RDC	8.4 (1.2)	1 vs 2: 0.54	7.6 (1.9)	1 vs 2: 0.49
2. CDC	8.5 (1.9)	2 vs 3: **0.045**	7.3 (2.6)	2 vs 3: 0.93
3. MF	7.6 (2.4)	1 vs 3: 0.06	7.2 (1.8)	1 vs 3: 0.33
Grindability of material				
1. RDC	8.7 (1.2)	1 vs 2: 0.86	8.7 (1.5)	1 vs 2: 0.30
2. CDC	8.4 (1.2)	2 vs 3: 0.10	8.3 (1.7)	2 vs 3: 0.95
3. MF	8.2 (2.1)	1 vs 3: 0.10	8.3 (1.7)	1 vs 3: 0.23

Significant *p* values are in bold

## Discussion

Based on the results, null hypothesis 1 can be accepted. However, null hypothesis 2 (equal failure dentist and student treatment) has to be rejected. Short-term clinical performance was satisfactory and comparable for all adhesively retained core build-up materials tested before placement of a permanent single crown or FDP. Early losses and failures were quite rare, particularly for patients treated by a dentist (3.3% failure). In general, the performance of the resin core build-ups was more favorable than that observed in previous studies. Approximately 8% of the resin core build-ups in our study failed before incorporation of the permanent fixed dental prosthesis, whereas Stober and Rammelsberg [18] calculated an early incidence of failure for composite materials of 15%. It is worth discussing the potential reasons for this difference. One reason might be that the majority (two-thirds) of participants in the 2005 study were treated by dental students. In our study, conversely, only half of participants were treated by dental students. Both our study and the previous study [18] found that the incidence of failure for resin core build-ups differed significantly between dentists and dental students, which is not surprising. Because the use of adhesively retained composite resin materials is particularly technique-sensitive, it might be assumed that a greater amount of clinical experience corresponds to a greater incidence of success. Dentists have more experience of tissue and moisture control than students do, particularly in relation to large and subgingival cavities. Another aspect which might have affected the differences in failure rates between the 2005 and the recent study is that other/further developed adhesive materials have been used, albeit the previous study already used composites in combination with total-etch technique [18].

The significantly higher incidence of failure found in our study for > 3 surfaces and for students as operators seems to suggest that more clinical experience does indeed equate to a higher incidence of success for this technique. Compared with the dentists, the dental students generally required more attempts to take an adequate impression and also changed temporary restorations more often in order to improve preparations, probably stressing the adhesive interface of the core build-ups to stump (although the effect of these two factors is not significant in this study). However, if only looking at the incidence of failure for restorations performed by dentists and only those that are adhesive (self-etch and etch-and-rinse) in nature (6–14% depending on the material), the outcome of our study is still more favorable than that of previous studies. Although Stober and Rammelsberg’s study [18] investigated both vital and non-vital teeth, adhesion to deep cavities of non-vital teeth can be negatively affected by alterations in the dentin structure (root dentin, secondary dentin, sclerosis, etc.). Therefore, only vital teeth were included in our study. In a different study on the short-term performance of core build-ups in vital teeth, Simons et al. actually observed no early failures [19]. However, the cavities in that study were on average smaller than those in both our study and the study by Stober and Rammelsberg [7], and it is unclear whether students, dentists, or both performed the treatments. Moreover, with regard to the size of core build-ups, the results of our study indicate that substantially more failures occur for core build-ups of > three reconstructed surfaces. This is because a larger core build-up results in a decreased adhesive interface. It is worth noting in this context that the adhesion of resin core build-up materials is often limited to the dentin surface after preparation for a full crown or FDP. Minerals in enamel and dentin are dissolved by phosphoric acid etching and a micro-retentive pattern is infiltrated by resin monomers. The hydrophilicity of the dentin and collagen fiber scaffold limits infiltration of dentinal tubules and adhesion. At the same time, as discussed above, larger or subgingival cavities might hinder absolute moisture control by a rubber dam, for example. It has been suggested that rubber dam isolation results in better bonds in restorative treatment [12], but this must be omitted for larger or subgingival cavities. In our study, a rubber dam could be used in 13% of cases only (in the ratio 1:5 for treatment by dentists and dental students, respectively). This results in a biased statistical evaluation, but the trend is strongly toward a lower incidence of failure for the use of rubber dams. Another finding worthy of discussion is that early incidence of failure did not differ between the two core build-up materials using the total-etch approach (Rebilda DC and Multicore Flow) and the material using the self-etch approach (Clearfil DC Core). Furthermore, because we only examined the early failure of build-ups before incorporation of the fixed dental prosthesis, the effect of problems such as long-term hydrolytic degradation and microleakage—which can be more pronounced if self-etch techniques are used—was not substantial [13, 14]. Even in the long term, the possible effect of such problems would be minor because the prosthetic restoration margins of the ferrule preparation extend into the natural tooth structure. One concern often raised in connection with the use of self-etch adhesives is the possible inhibition of self- or dual polymerization of the composite materials by bonding components, e.g., accelerators [21, 22]. However, the bonding agents and composite materials used in our study according to manufacturer instructions seem perfectly compatible [23], and no specific adverse events were observed. In light of this, the operator ratings were somewhat unexpected. Rebilda DC achieved slightly but significantly better scores for grindability and handling than Multicore Flow. Because all materials had similar dentin-like properties in a cured state, this calls the grindability rating into question. It should be mentioned in this context that Rebilda DC is the standard core build-up material used at our department and is therefore perhaps more familiar to the operators. In terms of handling properties, the difference between Rebilda DC Core and Multicore Flow might be explained by the fact that Multicore Flow requires one more preparation step (second adhesive: Heliobond) than Rebilda DC does. According to this line of thinking, a significantly better rating might have been expected for Clearfil DC, which is used with an all-in-one adhesive. However, this was not the case. Indeed, a study by van Landuyt et al. [14] did not consistently find an advantage for one-step adhesives in terms of user comfort. Interestingly, if only the dentists’ ratings are analyzed, Clearfil DC Core received a better handling score than Multicore Flow did. To this end, use of self-etch materials like Clearfil DC applied according to manufacturer’s instructions can provide advantages—especially in use for core build-ups where no or less enamel is remaining after preparation—compared to the total-etch technique. The one-step application is quicker and therefore, less contamination and errors can occur during the technically sensitive bonding procedure [13, 14]. The milder etching component of the self-etching adhesives reduces the risk of overetching of the dentin. Overetching, however, can evoke nanoleakage and worse adhesion reliability leading to post-operative pain.

### Strengths and limitations

The single-blind randomized controlled design of this study has a high exploratory power; however, drop-outs might have resulted in statistical bias. The drop-out incidence of approximately 10% is, however, within the range usually accepted in clinical studies. Furthermore, the number of core build-ups investigated for molars was greater than that for premolars and anterior teeth, which might have affected analysis of the variable “tooth region.” This distribution does, however, reflect the fact that FDP treatment is most commonly required for molars (distribution in addition affected by selection of study tooth according to FDI scheme). Furthermore, it is worth to discuss that only one cavity (largest coherent volume) was considered for the study. This method includes advantages and disadvantages. Inclusion of only one case per patient guaranties a high standard in view of statistical effects as clustering (negative or positive effects of the patient itself) can be avoided. Of course, the patient bias could alternatively be reduced by considering random effects covariates. However, inclusion of the largest cavity may lead to overestimation of failure as larger core build-ups are at higher risk for failure. One major weakness of the study is that the resin core build-up material Clearfil DC Core was replaced by a successor product (Clearfil DC Plus) during the study recruitment period. However, an additional sensitivity analysis showed that the incidence of failure did not differ significantly between the two materials (chi-squared tests; *p* = 0.971). The analysis of moisture control (rubber dam isolation vs relative moisture control, more frequent use of the first by dental students) and operators’ visual analog scale (VAS) ratings should be interpreted with caution because information regarding these was not provided by all operators (*n* cases missing = 8).

## Conclusions

The incidence of failure for adhesively retained composite resin core build-ups is manageable. The predominant risk factors for the loss of a core build-up are a larger cavity size and relative lack of clinical experience. In this study, the clinical success of resin core build-ups using self-etch adhesives appears comparable with that of materials processed using the total-etch technique, at least in the short term. Use of rubber dam—whenever possible—seems to demonstrate a trend to reduction of failures.
